# Inhibitors of the Fanconi anaemia pathway as potential antitumour agents for ovarian cancer

**DOI:** 10.37349/etat.2020.00003

**Published:** 2020-02-29

**Authors:** Sarah J Taylor, Mark J Arends, Simon P Langdon

**Affiliations:** Cancer Research UK Edinburgh Centre and Edinburgh Pathology, Institute of Genetics and Molecular Medicine, University of Edinburgh, Crewe Road South, EH4 2XU Edinburgh, UK; Istituto Nazionale Tumori “Fondazione Pascale” Via Mariano Semmola, Italy

**Keywords:** Fanconi anaemia protein, ovarian cancer, carboplatin, cisplatin, inhibitors, DNA repair

## Abstract

The Fanconi anaemia (FA) pathway is an important mechanism for cellular DNA damage repair, which functions to remove toxic DNA interstrand crosslinks. This is particularly relevant in the context of ovarian and other cancers which rely extensively on interstrand cross-link generating platinum chemotherapy as standard of care treatment. These cancers often respond well to initial treatment, but reoccur with resistant disease and upregulation of DNA damage repair pathways. The FA pathway is therefore of great interest as a target for therapies that aim to improve the efficacy of platinum chemotherapies, and reverse tumour resistance to these. In this review, we discuss recent advances in understanding the mechanism of interstrand cross-link repair by the FA pathway, and the potential of the component parts as targets for therapeutic agents. We then focus on the current state of play of inhibitor development, covering both the characterisation of broad spectrum inhibitors and high throughput screening approaches to identify novel small molecule inhibitors. We also consider synthetic lethality between the FA pathway and other DNA damage repair pathways as a therapeutic approach.

## Introduction

The Fanconi anaemia (FA) pathway is a DNA repair pathway that identifies and removes DNA interstrand cross-links (ICLs) within cells, which occur when opposing strands of the DNA double helix are connected together, preventing their separation and restricting replication and transcription [1, 2]. The pathway incorporates component steps of recognition and unhooking of ICLs, translesion synthesis (TLS), homologous recombination (HR) and nucleotide excision repair (NER). There is therefore extensive crossover between components of the FA pathway and other DNA damage repair pathways. The key players within the pathway, the FA proteins (Table 1), have been identified through genetic mutations resulting in loss of function of the pathway and hence susceptibility to Fanconi anaemia, a rare autosomal and X-linked genetic disease characterised by increased predisposition to bone marrow failure, congenital defects and cancer [1, 2].

**Table 1.** Fanconi anaemia protein family and associated proteins

**Approved gene nomenclature**	**Alias**	**Area of pathway**	**Role in ICL**
***FANCA***		FA core complex	Functions in the AG20 subcomplex with FANCG to promote FA core complex localisation and translocation
***FANCB***		FA core complex	Functions in the BL100 subcomplex with FANCL to improve efficiency of FANCD2/I ubiquitylation and provide structural scaffold
***FANCC***		FA core complex	Component of the CEF subcomplex with FANCE, FANCF to stabilize interactions between FANCD2/I complex and FA core complex and improve ubiquitylation efficiency
***FANCE***		FA core complex	Component of the CEF subcomplex with FANCC, FANCF to stabilize interactions between FANCD2/I complex and FA core complex and improve ubiquitylation efficiency
***FANCF***		FA core complex	Component of the CEF subcomplex with FANCC, FANCE to stabilize interactions between FANCD2/I complex and FA core complex and improve ubiquitylation efficiency
***FANCG***	*XRCC9*	FA core complex	Functions in the AG20 subcomplex with FANCA to promote FA core complex localisation and translocation
***FANCL***		FA core complex	Component of the BL100 subcomplex and E3 ubiquitin ligase controlling monoubiquitylation of FANCD2/I
***FANCM***		FA core complex	Binds FA core complex to chromatin at ICL sites
***FANCT***	*UBE2T*	FA core complex	E2 ubiquitin ligase controlling monoubiquitylation of FANCD2/I
***FANCD2***		FANCD2/I complex	Initiates unhooking by nucleases
***FANCI***		FANCD2/I complex	Initiates unhooking by nucleases
***SLX4***	*FANCP*	Unhooking	Recruits and regulates nuclease activity during unhooking
***ERCC4***	*FANCQ, XPF*	Unhooking	Key endonuclease mediating incision of crosslink during unhooking
***MAD2L2***	*FANCV, REV7*	TLS	Subunit of the TLS extension polymerase POLζ
***BRIP1***	*FANCJ, BACH1*	TLS/HR	Regulates pathway choice between TLS and HR repair
***BRCA2***	*FANCD1*	HR	Recruitment of RAD51 to ssDNA
***PALB2***	*FANCN*	HR	Mediates loading of BRCA1/2 complex on to single stranded DNA
***RAD51C***	*FANCO*	HR	RAD51 nucleoprotein filament assembly
***RAD51***	*FANCR*	HR	Forms nucleoprotein filaments which mediate template homology search and strand exchange
***BRCA1***	*FANCS*	HR	CMG helicase eviction, HR pathway promotion and TLS inhibition in complex with FANCJ, complex formation with BRCA2 during HR
***XRCC2***	*FANCU*	HR	RAD51 nucleoprotein filament assembly
***RFWD3***	*FANCW*	HR	Mediates RPA dynamics to promote HR
FAAP10^*^	*MHF2, CENPX, STRA13*	FA core complex	Localization of FANCM to chromatin
FAAP16^*^	*MHF1, CENPS, APITD1*	FA core complex	Localization of FANCM to chromatin
FAAP20^*^		FA core complex	Promotes stability of the AG20 subcomplex
FAAP24^*^		FA core complex	Associates with FANCM to mediate DNA binding of the FA core complex
FAAP100^*^		FA core complex	Functions within the BL100 subcomplex to provide structural scaffold

* Genes are not true FA family members as mutations have not been documented in FA patients, but are members of the family of FA Associated Proteins, and are required for the successful function of the FA pathway. *FAAP*: Fanconi anaemia associated protein

DNA repair pathways can act as a double-edged-sword in the context of cancer. While their loss, frequently through key mutations, results in increased genomic instability enhancing the likelihood of cancer developing in the first place, the lack of effective repair then allows certain chemotherapy drugs to be more effective. ICLs inhibit DNA replication and transcription unless repaired and hence are toxic to cells. Several anticancer drugs exploit this process, notably platinum-containing drugs such as carboplatin and cisplatin which are widely used to treat multiple cancer types including ovarian cancer. The platinum-containing drugs have become the leading first line chemotherapy to treat ovarian cancer and the majority of patients will initially respond to these agents [3]. However, in most cases, resistance will emerge largely driven by DNA repair processes and intense interest is currently focused on development of strategies that can inhibit these repair processes particularly in selected molecular subgroups. This has led to the development of poly ADP ribose polymerase (PARP) inhibitors (PARPis) which are most effective in BRCA-deficient ovarian cancers, in which context they exhibit a synthetic lethality effect [4]. It is feasible that a parallel strategy targeting components of the FA pathway may have value in stratified sub-groups of ovarian cancer patients and the focus and emphasis of this review will be on the potential use of FA inhibitors in this disease.

### The FA pathway in ICL repair

**Figure 1.**
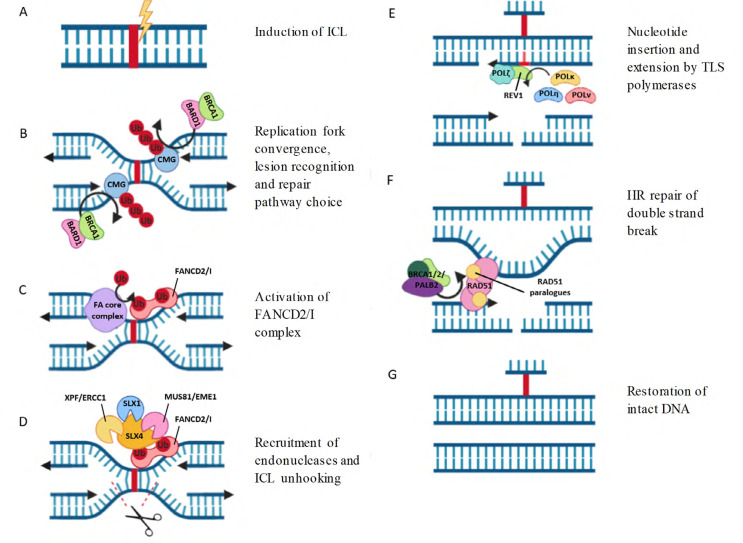
Repair of ICLs by the FA pathway. (A) ICLs are induced by platinum chemotherapy or other agents; (B) ICLs are recognised by converging replication forks. CMG helix is polyubiquitylated by TRAIP and removed by the BARD1/BRCA2 complex, allowing access to the ICL site; (C) the FANCD2/I heterodimer is recruited to chromatin and each subunit is monoubiquitylated by the FA core complex; (D) monoubiquitylated FANCD2/I recruits endonucleases, which create incisions in one DNA strand around the ICL site, unhooking the strands and generating a DSB; (E) insertion of a single nucleotide opposite the ICL by TLS polymerases, followed by strand extension by POLζ restores one DNA duplex; (F) the intact DNA duplex is used as a template for HR repair of the DSB. RAD51 is loaded on to chromatin by BRCA1/2/PALB2 complex and multimerizes with RAD51 paralogues to form protein nucleofilaments, enabling strand exchange and template directed extension; (G) intact DNA duplexes are restored. The bound ICL adduct is no longer a toxic block to replication and can be removed by NER. Figure created using Biorender

ICL repair occurs via two mechanisms—DNA replication dependent and independent repair—based on the cell cycle phase in which they occur. Replication dependent ICL repair is prevalent during S phase, when the presence of ICLs blocks progression of replication forks. This relies on the FA proteins for repair initiation, generating double strand breaks (DSBs) which are then repaired by HR [2] (Figure 1). Replication independent ICL repair on the other hand, occurs when ICLs prevent gene transcription in the G and S phases, does not involve the FA proteins, and resulting damage from lesion removal is repaired by NER [5]. Current knowledge of the interplay between these pathways is limited [6], although impairment of both produces an additive effect on cellular sensitization to cisplatin and mitomycin C (MMC), suggesting a degree of separation between the two [5]. This review will focus on the DNA replication dependent repair pathway, due to the strong links between the FA pathway and chemosensitivity in ovarian cancer [7], and the suspected prevalence of this repair mechanism in rapidly dividing cells, such as cancer cells, due to their pronounced intolerance for unrepaired ICLs in S phase [5, 8].

A secondary mechanism for DNA replication dependent ICL repair without the generation of double strand breaks involving the glycosylase NEIL3, without dependence on the FA proteins, has also been reported in cell free extracts in response to abasic site and psoralen induced ICLs [9]. FA protein mediated repair is however thought to be the major pathway involved in cisplatin induced repair [10], and the knockdown of FA proteins produces a more ICL sensitive phenotype than NEIL3 [11], making it the more relevant pathway for study in the context of cancer.

### Initiation of repair

When an ICL occurs (Figure 1A), this is initially sensed by the convergence of two replication forks at the ICL, which stall around 20 nucleotides from the damage site creating an *X* shape [12]. The unloading of the stalled CMG replicative helicase from DNA by the BRCA1-BARD1 complex is then triggered (Figure 1B). This prevents steric hindrance by the helicase, and provides a favourable structure for the binding of repair proteins [13]. This also allows one of the leading strands to subsequently proceed within one nucleotide of the ICL [12]. A recent study showed that pathway decision at this point is determined by the TRAIP E3 ubiquitin ligase, which initially monoubiquitylates CMG helicase, leading to the recruitment and attempted repair by NEIL3 [11]. In cases where NEIL3 is unable to repair the ICLs, such as those induced by cisplatin, TRAIP extends the length of the ubiquitin chains, resulting in CMG removal and enabling of the FA pathway progression [11].

### DNA binding of the FA complex and activation of the FANCD2/FANCI complex

**Figure 2.**
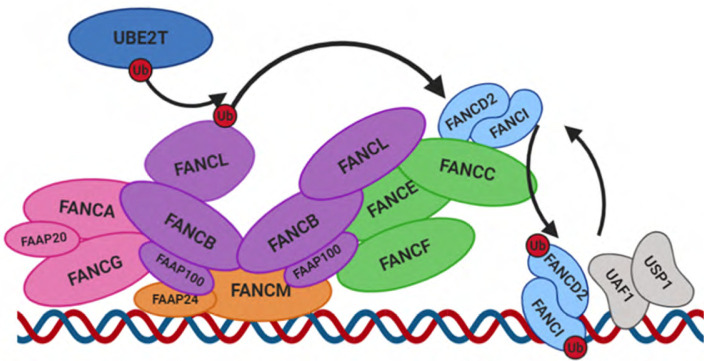
Monoubiquitylation of FANCD2/I by the FA core complex. The AG20 subcomplex (pink) is responsible for the translocation of the core complex to the nucleus. FANCM and FAAP24 associate to form a subcomplex (orange) anchoring the FA core complex to DNA at ICL sites. A central asymmetrical dimer of BL100 catalytic subcomplexes (purple) acts as an essential scaffold for the other subunits, and contains two FANCL molecules with distinct conformations. The FANCD2/I heterodimer (light blue) is bound by the FANCC/E/F subcomplex (green) and is monoubiquitylated by the E2 ubiquitin ligase UBE2T (dark blue) and corresponding E3 ligase FANCL via dynamic changes in the core complex conformation. On completion of ICL repair, the FANCD2/I heterodimer is deubiquitylated by the USP1/UAF heterodimer and dissociates from DNA. Figure created using Biorender

Following CMG helix removal, the FA core complex (Figure 2), which consists of FANCA, FANCB, FANCC, FANCE, FANCF, FANCG, FANCL, FANCM and UBE2T (FANCT) binds the ICL site [1] (Figure 1C). The translocation and accumulation of this within the nucleus is mediated by FANCA and FANCG, which form a sub-complex with FAAP20 within the core complex [14], and may also function as a scaffold to stabilize core complex assembly [15]. The loading of the core complex onto DNA is via FANCM, which functions in complex with the FA like proteins MHF1/2 (FAAP10 and FAAP16) and FAAP24, which stabilize its loading onto chromatin [16, 17]. There is evidence that the DNA binding affinity of FANCM is moderated by phosphorylation tied to the cell cycle, with moderate levels of phosphorylation associated with increased binding. This is consistent with the role of FANCM as the anchor responsible for FA complex binding to chromatin, and presents a method by which the binding of the complex and subsequent ICL repair is restricted to S phase [16].

The other components of the FA core complex also form discreet sub-complexes within the main complex, the main function of which is to ubiquitylate the FANCD2/FANCI heterodimer [18] (Figure 2). The key ubiquitin ligases behind this process are the E3 ligase FANCL, and exclusive corresponding E2 ligase UBE2T (FANCT), with FANCL co-ordinating ubiquitin transfer while UBE2T conveys substrate specificity [19]. Although UBE2T does associate with FANCL to enable effective ubiquitylation of FANCD2/FANCI, it is not required for the stability of the core complex, and is constitutively present on chromatin [20]. FANCL associates in a sub-complex with FANCB and FAAP100, known as the BL100 complex, two of which form a homodimer within the FA core complex [18, 21]. This homodimer provides the central structural scaffold to the core complex, besides orientating the two FANCL copies in different conformations at opposite ends of the complex and providing the molecular flexibility required for ubiquitylation to occur [22]. The distinct conformations of FANCL molecules indicates that these may play separate roles within the complex, potentially ubiquitylating different members of the FANCD2/FANCI dimer or aiding substrate binding [22]. While FANCL alone is sufficient to reconstitute monoubiquitylation of FANCD2 and FANCI *in vitro*, the orientation in which FANCL is bound by the other sub-complex components has been shown to be important to boost ubiquitylation efficiency of the FANCD2/FANCI complex. This allows for the co-ordinated monoubiquitylation of both FANCD2 and FANCI [18]. Two further identical sub-complexes consisting of FANCC, FANCE, and FANCF (CEF complex) mediate the interactions between the BL100 sub-complex and the FANCD2/FANCI heterodimer during ubiquitylation, altering its confirmation and stabilizing the interface between FANCD2 and FANCI to enable more effective ubiquitylation of both substrates [21]. Of note, the FANCD2/FANCI heterodimer has been recently shown to be recruited to stalled replication forks at ICLs prior to ubiquitylation via binding to methylated histone H4K20me2 [23], with DNA binding a requirement for effective monoubiquitylation to occur [24]. UHRF1/2 have also been implicated as early ICL sensors important for FANCD2 stimulation, with knockdown leading to reduction in FANCD2 recruitment and monoubiquitylation [25]. The ubiquitylation of both the FANCD2 and FANCI components is important to prevent the deubiquitylation of FANCD2 by the deubiquitinase USP1 and its binding partner UAF1 while it is DNA bound, as these cannot bind to the altered confirmation of monoubiquitylated FANCI, and thus cannot act on either subunit [16]. This implies a key regulatory function for monoubiquitylated FANCI, preventing premature pathway inactivation prior to ICL repair, and once FANCD2/FANCI has dissociated from DNA on completion, USP1/UAF1 is able to bind and deubiquitylate both FANCI and FANCD2 components [16]. Recent work has however challenged this theory, demonstrating a requirement for DNA, and the DNA binding activity of UAF1, for successful deubiquitylation of FANCD2/FANCI [26]. This deubiquitylation is essential for ICL repair completion [23]. However, the importance, timing and dynamics of dissociation of the other FA components in ICL is not yet understood.

### Recruitment of endonucleases and ICL unhooking

Once monoubiquitylated, the FANCD2/FANCI complex promotes the accumulation of nucleases at damage sites, which cleave the DNA strand either side of the adduct, unhooking it (Figure 1D). SLX4 (FANCP) is recruited to chromatin bound, monoubiquitylated FANCD2, where it binds via a UBZ domain and acts as a scaffold for recruitment of further nucleases [27]. These nucleases include the XPF/ERCC1 complex, MUS81/EME1 complex, and SLX1. Of these, the recruitment of XPF/ERCC1 appears to be the most important, as knockdown of XPF (FANCQ) induces a severe FA phenotype, and a minimal SLX4 peptide which interacts only with XPF/ERCC1 has been shown to be sufficient to restore ICL repair, with SLX4 enhancing the nuclease activity of XPF/ERCC1 100-fold by directing specificity to damage sites [28]. XPF/ ERCC1 is widely accepted to mediate initial DNA incision, with SLX4 stabilizing intermediate structures generated during repair [28]. The exonuclease SNM1A is also proposed to co-ordinate with XPF in this area of the pathway, possibly by processing of intermediate structures induced by XPF/ERCC1 [29]. FAN1, which has endo- and exonuclease activity, is also recruited to sites of DNA damage by interacting directly with monoubiquitylated FANCD2 via a UBZ domain, where it has been proposed to function in ICL unhooking [30], although this may be non-essential or have some redundancy with other nucleases, as it is not a true FA gene and knockdown conveys only partial sensitivity to ICL inducing agents [31]. While knockdown of any of the aforementioned nucleases have been shown to induce hypersensitivity to ICLs, only XPF and SLX4 are classified as FA proteins, and this part of the pathway remains poorly understood, with the length of excised DNA, repair intermediates generated following incision and how these are processed by nucleases remaining unknown [28]. As the replication fork is able to approach very close to the ICL, and a shorter DNA strand enhances the efficiency of downstream translesion synthesis (TLS), it is thought that the position of nucleolytic incision is likely very close to the ICL, and that a minimal section of DNA is excised [32].

The nucleolytic incision step essentially removes the ICL bound section of DNA backbone from one of the strands, separating the two connected DNA strands and generating unhooked intermediates. One strand remains bound to the section of DNA containing the ICL and a double strand break is induced in the second strand [33].

### Nucleotide insertion and extension by TLS polymerase

In the current model, the next stage in repair is insertion of a non-templated nucleotide opposite the ICL-bound base, thus allowing the bypass of the ICL and regeneration of an intact leading strand, albeit with the introduction of point mutations into the genome [34, 35] (Figure 1E). The TLS polymerases required for this step are specific to the structure of the DNA, and length and flexibility of the ICL, implying different polymerases may play roles in repair of ICLs induced by differing agents [32]. In the case of cisplatin induced adducts, it is speculated that multiple polymerases may function in ICL repair, and there may be some overlap in function, as while TLS polymerases POLκ, POLη and POLν have been shown capable of acting on ICLs, knockdown has conveyed at most a moderate increase in sensitivity to cisplatin, and not hypersensitivity [32, 33, 36]. POLν has also been shown to interact with FANCD2 and FANCI [37]. It has also been speculated that TLS polymerases may be nonessential to the process, and unhooked ICLs of short length may be bypassed entirely by replicative polymerases, as has been demonstrated in bacterial systems [38]. The exact process of lesion bypass by polymerase remains undefined.

REV1 and POLζ (which is composed of the REV3 catalytic subunit and REV7 (FANCW) accessory subunit), are also thought to be extremely important for this stage of ICL repair. Immunodepletion of REV7 results in a cisplatin hypersensitive phenotype [12], and it is classified as an FA gene [39]. It has been shown that REV7 can interact with both REV3 and REV1, which is essential for cisplatin resistance [40], and knockdown of these 3 proteins individually and together have been shown to produce very similar phenotypes, including hypersensitivity to ICLs [41], implying that these proteins co-operate to carry out an essential function in the FA pathway. REV1 is recruited to the ICL site by binding to the UBZ4 domain of FAAP20, a component of the FA core complex, an interaction that has been shown to be vital for REV1 foci formation and stimulation of TLS and which is enhanced by monoubiquitination of REV1 [42]. The presence of PCNA at the replication fork is also thought to play roles in the recruitment of TLS polymerases [43]. While it was once considered to be a main candidate as an essential TLS polymerase, nucleotide insertion by REV1 at cisplatin ICLs has been shown to be very low efficiency [32]. As REV1 has been shown to interact directly with other TLS polymerases, this may imply that, although it may not be directly responsible for the insertion step, it may play further roles in recruiting and co-ordinating the other TLS polymerases [44, 45]. POLζ on the other hand is thought to be essential for the extension of the leading strand beyond ICLs in distorted DNA following initial nucleotide insertion [12, 46], and this is dependent on REV1, giving further evidence for a role for REV1 in polymerase recruitment [47]. POLη has been previously shown to synergise with POLζ in binding DNA at cisplatin ICLs *in vitro*, and it has been proposed to mediate the efficient insertion of nucleotides enabling extension to occur [48], however more work is required to confirm this mechanism, and indeed to investigate why hypersensitivity to ICLs is not incurred by knockdown.

### Homologous recombination repair of double strand breaks

Following incision and extension of the parental DNA strand, one of the duplexes is restored, allowing this to function as a template for HR repair of the double strand break in the second strand (Figure 1F). Due to the requirement for HR in ICL repair downstream of the FA core complex, several key HR proteins are also classified as Fanconi anaemia genes. FANCJ (BRIP1), a DNA helicase, is thought to be responsible for mediating the switch from TLS to HR processing. When bound to PMS2 and MLH1 of the mismatch repair complex, HR is inhibited, and TLS is promoted, in a POLη dependent manner [49, 50]. However when FANCJ is phosphorylated, it forms a complex with BRCA1 (FANCS), inhibiting TLS and stimulating HR [50]. BRCA1 and BRCA2 bind the site of the DSB in complex with PALB2 (FANCN), with PALB2 mediating the loading of the complex on to single stranded DNA [51]. BRCA2 recruits RAD51 to RPA coated ssDNA at damage sites, which enables RAD51 to oligomerize and form nucleoprotein filaments [52, 53]. Mediation of RPA dynamics at the strand are performed by the FA protein RFWD3 (FANCW), which ubiquitylates RPA at stalled replication forks, promoting HR [54]. The RAD51 paralogs, including the FA proteins RAD51C (FANCO) and XRCC2 (FANCU), are all required for formation of RAD51 filaments, and likely play roles in mediating their assembly, although exact roles have not yet been elucidated [55]. Recent work suggests that the paralogs function to induce structural changes in the RAD51 nucleoprotein filament, promoting and stabilizing an open, flexible conformation which is favourable for strand exchange [56]. This is essential for initiation of HR, stimulating homology search and strand invasion into the intact sister chromatid to provide a template for HR [57]. BRIP1 (FANCJ) has been shown to be capable of inhibiting RAD51 mediated strand exchange, and may function in its HR inhibitory role by displacing RAD51 from ssDNA [58]. RAD51 has also been shown to function outside of its canonical role in HR during ICL repair, protecting DNA at the stalled replication fork from nonspecific degradation and unwinding [59]. Completion of HR is the final step in ICL repair, as two intact duplexes are restored and there is no longer any barrier to DNA replication or transcription. While the excised cisplatin still remains bound to a single strand, this is no longer a toxic block to replication, and can be removed at a later stage, likely by NER [60] (Figure 1G).

### The FA pathway in ovarian and other cancer types

Due to the overwhelming predisposition for cancer development in FA patients [61], the role of this pathway has been extensively studied in a cancer context. Suppression of the FA pathway increases genomic instability by impairing DNA damage repair and allowing the incorporation of more errors into the genome, leading to chromosome breakage and accumulation of a higher mutational burden. FA patients tend to carry biallelic germline mutations, except in rare cases of X-linked *FANCB* and dominant negative *RAD51* mutated patients [62]. This leads to the increased formation of tumours, predominantly acute myeloid leukaemia (and its precursor myelodysplastic syndrome), squamous cell carcinoma (oral, oesophageal and vulval), hepatocellular carcinoma and others [63]. Distinctions also exist between patients with mutations in different FA complementation groups, for example those with *BRCA1/2* biallelic mutations are predisposed not only to AML, but also brain tumours [62]. While monoallelic mutations tend not to cause full FA, they have been linked to increased risk of cancer for several FA proteins, for example germline mutations in *BRCA1/2* lead to increased familial breast and ovarian cancer risk [64]. Somatic mutations and other silencing mechanisms of the FA genes have also been observed in cancers associated with FA arising in non-FA patients. For example silencing of *FANCA*, which accounts for two thirds of FA cases and is strongly associated with increased AML risk, has been observed in spontaneously occurring AML [65]. In contrast, in spontaneous ovarian cancers *FANCA* mutations are rare, with both germline and somatic mutations in *BRCA1/2* occurring far more frequently than any other FA genes [66]. This implies that, while they function in the same pathway, alterations in different FA proteins can be significant in different ways, especially in alternative cellular contexts.

The FA pathway is therefore frequently implicated as a tumour supressing mechanism [63]. Following the initial development of tumours, however, selective pressures can also lead to the re-expression of the FA proteins as a survival mechanism [7].

Standard of care platinum based chemotherapeutics induce both intrastrand crosslinks and ICLs, with ICLs thought to be the main adduct responsible for the toxic effects despite accounting for only 5–10% of adducts. This is due to their ability to cause extreme distortion in the DNA double helix structure, blocking key cellular processes such as DNA replication and gene expression [67, 68]. Indeed, in cisplatin resistant ovarian cancer cells, increases in ICL repair have been reported while intrastrand crosslink repair remains unchanged [69], and increased ICL repair in response to platinum chemotherapy in patients has been observed [70]. The FA pathway has been shown to be at least partially responsible for this, with key FA genes upregulated following exposure of cells to cisplatin [71], and inhibition of the pathway leading to resensitization [72]. Further, it has been shown that during the development of chemoresistant cell lines, FA pathway deficient cancer cells are able to adopt reversal mutations and demethylate promoter sites of FA genes to restore pathway function and promote survival [7, 73].

Aside from the documentation of the FA pathway in general in ovarian cancer, particularly chemoresistance, most of the individual pathway components have also been linked in various ways to a wide range of cancers. This singling out of specific proteins enables the identification of which parts of the pathway may provide the best druggable targets for development of therapeutic inhibitors.

### BRCA1 and BRCA2

The roles of *BRCA1* and *BRCA2* mutations in chemosensitizing ovarian cancer and improving prognosis are well known [74, 75], as are the mechanisms by which these can be reversed in development of chemoresistance [73]. Indeed, studies of BRCA1/2 have led to the development of Olaparib, a PARP inhibitor which has demonstrated impressive improvements in progression free survival in *BRCA* mutated patients [76]. As the BRCA story has been thoroughly documented in many previous reviews this review will instead focus on the role of the less well-known FA proteins.

### FA core complex proteins

*FANCF* has been demonstrated to be supressed by gene hypermethylation in ovarian cancer, leading to a chemosensitive phenotype which is reversed by demethylation during the development of chemoresistance [7]. *FANCF* deficiency has been linked to development of ovarian cancer *in vivo* [77], and methylation has been observed as a mechanism of suppression in patient samples [78].

A role for FANCA is well characterized in ovarian cancer chemoresistance. FANCA has been shown to be upregulated in response to various chemotherapeutic treatments in ovarian cancer spheroids [79], and knockdown has resensitized resistant breast cancer cell lines to cisplatin [80]. Deficiency has also been shown to sensitize ovarian cancers to PARP inhibitors, demonstrating the case for synthetic lethality of FA proteins other than BRCA1/2 with PARP inhibitor treatment [81].

The FA catalytic core is also of great interest for targeted inhibitors. Knockdown of both FANCL and FANCB in chicken cells has been documented to produce more profound sensitivity to mitomycin C, a compound which induces ICL formation in cells, than knockdown of other components of the FA core complex [82]. However, despite the key roles this sub-complex plays in the FA pathway, there are limited studies linking these proteins to tumourigenesis. So far, pathogenic mutations in *FANCL* have been shown to predispose to pancreatic cancer [83], and *FANCL* silencing has been linked to chemosensitization in lung cancer [84]. FANCB expression has no published data to date linking it to cancer, only excluding its involvement as a hereditary factor in breast cancer development [85].

The E2 ligase UBE2T (FANCT) has not been linked with chemoresistance in ovarian or other cancer types and is a recently categorised FA protein [86]. It is associated with ICL repair, and knockdown in amoeba has been shown to moderately sensitize them to cisplatin [87]. Drug screening has identified disruption of the interaction between UBE2T and FANCL as a chemosensitizing event in U20S cells treated with cisplatin [88]. Amplification has been linked to poor prognosis in breast and lung cancers [89] and hepatocellular carcinoma [90]. UBE2T also appears to have multiple functions outside the FA pathway, with knockdown causing decreased proliferation, invasion and migration as a result of AKT signalling suppression in osteosarcoma and nasopharyngeal carcinoma [91, 92], and increased apoptosis and cell cycle arrest in bladder cancer [93].

*FANCG* mutation has been reported to cause sensitivity to cisplatin in pancreatic cancer cell lines [94]. A study in ovarian cancer has identified increases in *FANCG* mRNA expression associated with the acquisition of a chemoresistant phenotype in ovarian cancer cells [95].

Another sub-complex within the main core complex consists of FANCC, FANCE and FANCF. Mutations have been observed in *FANCE* which have been speculated to facilitate the development of colorectal cancer [96], and *FANCE* alternative splicing may impair ICL repair in breast cancer [97]. However, another study could not find a significant link between *FANCE* mutation and breast cancer [98]. Deletion and hypermethylation of *FANCC* have been associated with breast cancer development, and surprisingly given the chemosensitizing properties of other FA pathway impairments, are associated with poor patient prognosis [99, 100]. Indeed, in hepatocellular carcinoma, lung and bladder cancer, silencing of *FANCC* is associated with improved chemosensitivity and response to treatment [101–103].

While whole exome sequencing has identified mutations of *FANCM* as a high risk factor for ovarian and breast cancers [104, 105], and another study has demonstrated increased cancer risk and chemotherapy toxicity in individuals with *FANCM* mutations [106], mutations appear to be less pathogenic than those in the other FA proteins [107]. *FANCM* knockdown has been shown to confer only partial disability of the FA complex and moderate sensitization to DNA crosslinking, due to overlapping functions with FAAP24, although the two do have some non-overlapping functions and so do not function redundantly [10]. This would however decrease the likelihood of FANCM playing a key role in FA pathway changes, and limit its utility as a target for inhibition.

### FANCD2 and FANCI complex

FANCD2 is a highly characterised component of the FA pathway, of particular interest due to its ubiquitylation status as a marker of pathway activation. Low expression of FANCD2 has been linked to development of ovarian cancer and enhanced sensitivity to therapy [108, 109]. Loss of expression has been noted in 10–20% of breast cancers, and high expression is correlated with poor outcome [110]. FANCD2 expression has been proposed to be upregulated by mTOR pathway signalling in leukaemia [111, 112], and the TIP60 translation factor in nasopharyngeal cancer [113] as a mechanism of resistance to platinum therapies.

Knockdown of the FANCI protein, which functions alongside FANCD2 in the FANCD2/FANCI complex, has been associated with enhanced cisplatin sensitivity in amoeba [87], although only limited studies of its role in cancer have been performed to date, with one study linking it to an aggressive phenotype in pancreatic cancer [114].

### Unhooking and TLS FA proteins

*SLX4* (*FANCP*) mutations have been observed in breast cancer, however it has been determined in multiple studies that this is not frequent enough to class *FANCP* as a susceptibility gene [115–117]. Knockout in mice predisposed them to epithelial cancer development, and confers sensitivity to DNA cross linkers, leading to the description of *SLX4* as a tumour suppressor [28]. *SLX4* mutations have also been observed across a panel of cancer cell lines, with pathogenic mutations associated with poor response to treatment with DNA damaging agents [118].

Overexpression of the XPF-ERCC1 complex has been implicated in ovarian cancer chemoresistance [119], and XPF overexpression in xenograft mice has been associated with poor prognosis and limited response to chemotherapy [120]. REV7 has been shown to be frequently expressed in ovarian cancer, with expression associated with poor prognosis and knockdown causing platinum sensitivity both *in vivo* and *in vitro* [121].

Mutations in *BRIP1* have been shown to confer high risk of prostate, breast and ovarian cancer [122, 123]. High expression has been linked to chemotherapy resistance and poor outcomes in gastric and colorectal cancers [124, 125], although conflicting results have been observed in cervical cancer, with overexpression causing sensitization of xenograft tumours to cisplatin, possibly due to causing inhibition of Rac1 signalling [126]. Based on the literature evidence, BRIP1 may be expected to be of interest in targeted therapy development.

### Homologous recombination FA proteins

There is extensive information on the roles of *BRCA1* and *BRCA2* as tumour suppressor genes in ovarian cancer and this review will instead describe the available information on the other FA proteins involved in HR.

RAD51 attenuation by miRNA has been shown to sensitize ovarian tumours to cisplatin and PARP inhibition *in vivo*, improving progression free survival [127]. *PALB2* (*FANCN*) is a documented breast cancer susceptibility gene [128], with disruption of the interaction between it and BRCA1 thought to be the key driver of this [129]. There is also evidence that *PALB2* silencing by mutation and DNA hypermethylation predisposes individuals to ovarian cancer [66, 130]. The role that PALB2 plays in ovarian cancer chemoresistance has not yet been documented, although mutation has been associated with sensitivity to DNA damage in pancreatic tumours [131]. Given its close association with the *BRCA1/2* genes and HR, it would be unsurprising if dynamic changes in expression with chemotherapy treatment were observed, and it is unexpected that its role in ovarian cancer chemoresistance has not already been further characterised, particularly as mutations have been shown to sensitize sarcoma tumours to PARP inhibitors [132]. Investigation of PALB2 may therefore aid in patient stratification for PARP inhibitor treatment.

XRCC2 (FANCU) was only recently classified as a FA protein due to mutations conferring sensitivity to ICL inducing agents [133]. The promoter has been reported to be hyperactivated in many cancer types, with attenuation of this *in vivo* slowing tumour growth [134]. XRCC2 overexpression has also been identified as a marker for radioresistance, with knockdown causing enhanced sensitivity [135]. Mutation was initially associated with breast cancer development [136], however this has recently been disputed, and remains controversial [137, 138]. Knockdown of XRCC2 is also known to cause sensitivity to PARP inhibitors [139].

*RAD51C* (*FANCO*) has been shown in multiple studies to be an ovarian cancer susceptibility gene [140, 141]. However, these studies looked only at the effect of mutations. Promoter methylation may be involved in downregulation, which is reported to occur in 2% of cases, so there may be additional factors altering expression. The same study also found that *RAD51C* silencing was associated with sensitivity to PARP inhibitors in ovarian cancer [142], confirming that other members of the FA/HR pathway may also have utility as patient stratification biomarkers. Similar to BRCA1/2, in cases where pathogenic mutations in RAD51C sensitize tumours to PARP inhibition, resistance mechanisms via the acquisition of secondary mutations have been reported [143]. High expression has also been shown to predict poor patient survival and resistance to cisplatin therapy in lung cancer [144].

RFWD3 (FANCW) knockdown increases sensitivity to DNA damaging agents [145], and increased expression following DNA damage in gastric cancer has been shown [146], although little other information exists linking it to cancer. It may therefore merit further study.

## Current state of targeted therapies

Due to the critical roles that the FA pathway has been reported to play in chemoresistance across a wide range of cancers, it clearly makes an appealing target for inhibition by targeted therapies to enhance the effectiveness of chemotherapy. However, despite the long association between the FA pathway and response to chemotherapy, the development of such drugs has made slow progress. Only in recent years have studies begun to investigate more closely the potential of small molecule inhibitors of this pathway (Table 2). Aside from PARP inhibitors, which were initially designed with the HR pathway in mind, none have yet progressed to clinical trials in man.

**Table 2.** Inhibitors of the Fanconi anaemia pathway

	**Target**	**Broad /specific**	**Mechanism of action**	**Synergy with chemotherapy**	**Direct Binding shown**	**ICL inhibition demonstrated**	**Cancer type investigated**	**Part of pathway inhibited**	**Reference**
**Curcumin**	Unknown	Broad	Proteasome and kinase inhibitor	Cisplatin, not paclitaxol	No	FANCD2 monoubiquitylation and foci	Ovarian	FANCD2/I activation	[147]
**Wortmannin**	Unknown	Broad	Kinase inhibitor	No	No	FANCD2 monoubiquitylation and foci	Ovarian	FANCD2/I activation	[72, 146]
**H-9**	Unknown	Broad	Kinase inhibitor	No	No	FANCD2 monoubiquitylation and foci	Ovarian	FANCD2/I activation	[72, 147]
**Alsterpaullone**	Unknown	Broad	Kinase inhibitor	No	No	FANCD2 monoubiquitylation and foci	Ovarian	FANCD2/I activation	[72, 147]
**DDN**	Unknown	Broad	Unknown	Cisplatin	No	FANCD2 monoubiquitylation and foci	Ovarian	FANCD2/I activation	[149]
**Bortezomib**	Unknown	Broad	Proteasome inhibitor	Cisplatin	No	FANCD2 monoubiquitylation and foci, RAD51 foci	Ovarian	FANCD2/I activation	[72]
**17-AAG**	Unknown	Broad	HSP90 inhibitor	Cisplatin	No	FANCD2 monoubiquitylation and foci, RAD51 foci	Ovarian	FANCD2/I activation	[72]
**CA-074-Me**	Unknown	Broad	CathepsinB inhibitor	Cisplatin	No	FANCD2 monoubiquitylation and foci, RAD51 foci	Ovarian	FANCD2/I activation	[72]
**Compound 7012246**	Unknown	Broad	Unknown	Cisplatin	No	FANCD2 monoubiquitylation and foci, RAD51 foci	Ovarian	FANCD2/I activation	[72]
**Compound 5373662**	Unknown	Broad	Unknown	Cisplatin	No	FANCD2 monoubiquitylation and foci, RAD51 foci	Ovarian	FANCD2/I activation	[72]
**Gö6976**	Unknown	Broad	PKC, CHK1 inhibitor	Cisplatin	No	FANCD2 foci, RAD51 foci	Ovarian	FANCD2/I activation	[72]
**SB218078**	Unknown	Broad	CHK1, CDC2, PKC inhibitor	Cisplatin	No	FANCD2 foci, RAD51 foci	Ovarian	FANCD2/I activation	[72]
**UCN-01**	Unknown	Broad	PKC, CHK1, CDK, AKT inhibitor	Cisplatin	No	FANCD2 monoubiquitylation and foci, RAD51 foci	Ovarian	FANCD2/I activation	[72]
**Geldanamycin**	Unknown	Broad	HSP90 inhibitor	Cisplatin	No	FANCD2 monoubiquitylation and foci, RAD51 foci	Ovarian	FANCD2/I activation	[72]
**Chloroquine**	Unknown	Broad	Lysosome and drug pump inhibition	Cisplatin	No	FANCD2 foci, RAD51 foci	Ovarian	FANCD2/I activation	[72]
**Puromycin**	Unknown	Broad	Protein Synthesis inhibitor	Cisplatin	No	FANCD2 monoubiquitylation and foci, RAD51 foci	Ovarian	FANCD2/I activation	[72]
**EF24/4H-TTD**	IKK	Broad	IKK inhibitor	MMC	No	FANCD2 monoubiquitylation and foci	Cervical	FANCD2/I activation	[148]
**Ouabain**	p38 kinase	Broad	Inhibitor of MMC induced S-phase arrest	MMC	No	FANCD2 monoubiquitylation and foci, FANCD2/FANCI mRNA	Osteosarcoma	FANCD2/I activation	[150]
**MLN4924**	NAE1	Specific	Proteasome inhibitor	MMC	No	FANCD2 monoubiquitylation and foci	Ovarian, cervical	FANCD2/I activation	[151]
**PIP-199**	RMI/FANCM	Specific	Inhibition of protein-protein interaction	Unknown	Yes	None	None	FANCD2/I activation	[152]
**CU2**	UBE2T/FANCL	Specific	Inhibition of FANCD2 monoubiquitylation by FANCL	Carboplatin	No	FANCD2 monoubiquitylation and foci	Osteosarcoma	FANCD2/I activation	[88]
**E-X PPI2**	ERRC1/XPF	Specific	Inhibition of protein-protein interaction	Cisplatin	Yes	None—NER focus	Melanoma, ovarian	Unhooking	[153]
**E-X AS5-4**	ERRC1/XPF	Specific	Active site inhibitor	Cisplatin	Yes	None—NER focus	Melanoma	Unhooking	[153]
**E-X AS5-7**	ERRC1/XPF	Specific	Active site inhibitor	Cisplatin	No	None—NER focus	Melanoma	Unhooking	[153]
**Compound 13**	ERRC1/XPF	Specific	Active site inhibitor	Cisplatin	Yes	None—NER focus	Melanoma	Unhooking	[154]
**Compound 7**	REV7/REV3L	Specific	Inhibition of protein-protein interaction	Cisplatin	Yes	None	Cervical	TLS	[155]
**Halenaquinone**	RAD51	Specific	Inhibition of RAD51-dsDNA interaction	Unknown	Yes	RAD51 homologous pairing	None	HR	[156]
**IBR2**	RAD51	Specific	RAD51 degradation by proteasome	Imatinib	Yes	RAD51 foci, HR	Chronic myeloid leukaemia	HR	[157]
**B02**	RAD51	Specific	Inhibition of RAD51-DNA interaction	Cisplatin (also *in vivo*)	Yes	RAD51 foci	Breast	HR	[158, 159]
**RI-1**	RAD51	Specific	Destabilization of RAD51 oligomerization	MMC	Yes	RAD51 foci	Breast, cervical, osteosarcoma	HR	[160]

### Inhibitors of FANCD2 monoubiquitylation

While few studies have aimed at development of specific inhibitors for the initial part of the pathway, the mediation of FANCD2 monoubiquitylation by the FA core complex, several broad spectrum inhibitors have been identified with the capacity to inhibit this monoubiquitylation, and thus disrupt pathway function. The earliest of these studies utilized a cell based assay to monitor formation of fluorescently labelled FANCD2 foci within the nucleus in response to commercial compound libraries. This identified curcumin, and three protein kinase inhibitors wortmannin, H-9 and alsterpaullone as inhibitors of FANCD2 foci formation, and mediators of cisplatin sensitivity [147]. A further study sought to improve upon the efficacy of curcumin as an ICL inhibitor by assessing monoketone analogues, and identified EF24 as having greater specificity and activity against FANCD2 monoubiquitylation in cell free xenopus extracts. A commercially available compound 4H-TTD, with structural similarity to EF24, was also identified as having similar effects. Importantly, these experiments also proposed a mechanism of action for curcumin and EF24, in targeting IKK, a major component of the NF-κB signalling pathway, which has been documented to interact with the FA core complex [148]. Further screening of chemical compound libraries using the same xenopus system also identified 2,3-dichloro-5,8-dihydroxy-1,4-naphthoquinone (DDN) as an inhibitor of FANCD2 monoubiquitylation. The effect of DDN on the FA pathway was confirmed using isogenic FANCF deficient and proficient ovarian cell lines, in which DDN sensitized the proficient cells to cisplatin to a greater extent than the deficient line [149]. A more recent cell based chemical library screen showed that the cardiac glycoside Ouabain, used to treat heart failure and previously reported to reduce proliferation in various cancer cell lines, also inhibits monoubiquitylation of FANCD2 and sensitizes cells to mitomycin C through a p38 dependent mechanism, although the dependence of this on the FA pathway has not been demonstrated [150]. Inhibition of the Nedd8 system using MLN4924 has also been shown to be effective at indirectly targeting the FA pathway and inducing sensitivity to ICLs [151].

For the development of specific FA targeting drugs, one of the most obvious points for pathway inhibition is the critical monoubiquitylation event of FANCD2 by FANCL and UBE2T. Although few inhibitors of E2 enzymes exist due to their lack of deep binding pockets, initial work towards development of a UBE2T inhibitor appears promising. Structural studies combined with fragment library screening have identified an allosteric binding site, which can be bound by small molecule fragments leading to inhibition of substrate ubiquitylation. Although these fragments have low binding affinity, and thus have limited therapeutic potential themselves, they could be a good initial starting point for inhibitor development in future [161]. Another pilot study has identified the interaction between FANCM and the RecQ-mediated genome instability protein (RMI) complex, which prevents sister chromatid exchange events during ICL repair, and disruption of which results in cellular sensitivity to ICLs [162]. This interaction is dependent on the binding pocket formed by the RMI complex, therefore presenting a target for competitive inhibition by small molecules. Several molecules were identified to inhibit this interaction via fluorescence polarization screening of compound libraries, and one of these, PIP-119, was confirmed to bind directly to the RMI core complex [148]. While neither of these strategies have been yet tested in a cellular context, both represent good starting points for inhibitor development, and uncover mechanisms of inhibition which may be exploited by later studies.

Recently, the first drug to directly target FANCD2 monoubiquitylation by the FA core complex has been reported. Using a high throughput biochemical screen to measure the ubiquitylation of the E1 ligase UBE1, E2 ligase UBE2T and the RING domain of the E3 ligase FANCL which are responsible for the ubiquitylation of FANCD2, molecules from a compound library which inhibited the ubiquitylation of FANCL were identified. These were further tested for selectivity and efficacy in the cellular environment. Molecule CU2 was identified as a selective inhibitor of FANCL ubiquitylation by UBE2T, with low cytotoxicity to cell lines and high synergy with carboplatin treatment. While further work is required to elucidate a mechanism of action, it is proposed to bind the FANCL RING domain, preventing interaction with the E2 ligase [81].This development represents an exciting starting point for specific FA targeting inhibitors which may be further adapted to improve potency, and could lead to a new class of mechanistically characterised inhibitors which can be more easily translated to the clinic for treatment of chemoresistant cancers than current broad spectrum inhibitors.

### Broad spectrum inhibitors of the FA pathway

A more comprehensive study of nonspecific inhibitors of the FA pathway was carried out by Jacquemont et al. [72], in 2012. This aimed not only to identify further broad spectrum inhibitors of the FA pathway, but to characterise these beyond their ability to inhibit formation of FANCD2 foci and FANCD2 monoubiquitylation. This included ability to form RAD51 foci in response to ionizing radiation and chemotherapy (an indication of pathway activation downstream of FANCD2 foci formation), HR proficiency, proteasome activity, and synergism with cisplatin in both FA deficient and proficient isogenic ovarian cancer cell lines. Of 16,000 chemicals tested in cell lines, 26 were identified as FA pathway inhibitors, and 11 of these synergised with cisplatin treatment in ovarian cancer cells, with 9 displaying greater efficacy in FA proficient cells, indicating the importance of the FA pathway targeting functions. In addition to discovery of FA inhibitors, this study also identified new classes of molecule, such as CHK1 and HSP90 inhibitors, which could be repurposed as chemosensitizing drugs that act on the FA pathway. It also focuses on the clinical applications of these inhibitors, such as in the context of chemoresistant ovarian cancer [72].

Several of these inhibitors have also been tested in glioblastoma cell lines and primary cultures, in combination with alkylating chemotherapeutic agents, as is standard of care for glioma. While 4H-TTD and Ouabain produced excessive toxicities in cell lines even at low concentrations, curcumin, EF24 and DDN all demonstrated the capacity to chemosensitize cells in an FA dependent manner [163]. This would suggest that further preclinical studies of such inhibitors are required before they can progress to clinical trials. The lack of definition around the mechanisms of action of these broad spectrum compounds is also a significant barrier to their incorporation into clinical trials. The aforementioned inhibitors also appear to target the FA pathway in an indirect manner, without interaction with the proteins themselves. Therefore, more recent studies are taking a more targeted approach to designing drugs against specific pathway members and interactions.

### Inhibitors of downstream components of the FA pathway

The FA proteins which function downstream of FANCD2 monoubiquitylation are also of interest as targets for inhibition, particularly as they are often involved in multiple DNA repair processes. The complex of XPF and ERCC1, which function as a key endonuclease in both ICL repair and nucleotide excision repair (NER), has been of great interest in the search for targeted therapies. Initial high throughput endonuclease activity screens identified the N-hydroxyimide and catechol classes of molecules as inhibitors of ERCC1/XPF, which could then be improved by structural engineering [154, 164]. While there were initial selectivity issues with *N*-hydroxyimides, which preferentially inhibited an alternative endonuclease, flap endonuclease 1 (FEN1), a scaffold hop to a hydroxypyrimidinone core allowed the tuning of selectivity away from FEN1 and towards ERCC1/XPF. However, while the final compound was shown to directly bind ERCC1/XPF and had favourable ADMET properties, it failed to sensitize melanoma cells to cisplatin [164]. Although the candidate catechol was shown to sensitize cells to cisplatin, and direct binding with ERCC1/XPF was demonstrated, toxicity at higher drug concentrations was observed, with only a small effect on chemosensitivity observed at nontoxic doses [154] and there are concerns about the ability of off target effects caused by catechols to produce misleading results [165]. Therefore, these molecules may not demonstrate ideal inhibitor starting points, as many further improvements to them would be required. Another study identified initial hits capable of inhibiting endonuclease activity of ERCC1/XPF, then refined these to identify compounds which directly bind the endonuclease active site or the binding pocket of XPF required for the formation of the complex. These have also been shown to have good selectivity and potency, and sensitize melanoma cells to cisplatin [153], and they remain the most promising inhibitor leads developed against ERCC1/XPF so far. More recently, new tools have been developed which may further aid identification of ERRC1/XPF inhibitors. Computational methods have been used to map the active site of XPF, and propose novel inhibitors. These also provide insights for rational inhibitor design to improve existing compounds [166]. New high throughput screening methods for ERCC1/XPF have also been developed, using more biologically relevant components than current methods, which may improve robustness of screening hits and facilitate drug discovery in future [167].

### Homologous recombination inhibitors

Due to its central role in HR, a pathway which cancers frequently become addicted to, RAD51 has also unsurprisingly been a target for inhibitor studies. Screening of extracts from marine sponges identified halenaquinone, which inhibits the ability of RAD51 to perform homologous pairing by directly binding RAD51 and preventing it from binding to dsDNA. In cell lines, halenaquinone inhibited formation of RAD51 foci, although there is no data indicating chemosensitization or inhibition of ICL repair specifically [156]. A second study performed a larger library based screen, identifying compound RI-1 which prevents formation of RAD51-DNA nucleofilaments by direct covalent binding of the RAD51 surface that acts as an interface between protein subunits. This similarly impaired RAD51 foci formation, and was also shown to sensitize cancer cells to mitomycin C [160]. As both of these molecules unfortunately have Michael acceptor activity, which limits stability and can cause off target effects and toxicity in biological systems, a further study attempted to improve upon these by optimizing the structure activity relationship of RI-1. The new compound maintains the mitomycin C sensitization and RAD51 foci disruption properties of RI-1, but lacks Michael reactivity and binds RAD51 at the same site by a reversible, noncovalent mechanism. Although it is less potent than RI-1, the pharmacological properties make it a better candidate for drug development [168]. An independent screen identified another direct, specific inhibitor of RAD51 multimerization, IBR2, which functions via a different mechanism, disrupting binding to BRCA2 and mediating degradation of RAD51 via the proteasome. This was successfully used to resensitize leukaemia in both cell lines and mouse models in which resistance to tyrosine kinase inhibitors had developed, resulting in significant *in vivo* survival improvements with minimal toxicities [157]. Further structure activity relationship studies have improved the potency of this drug 5-fold in triple negative breast cancer, although more work remains to identify an effective therapeutic dose with minimal toxicity to facilitate clinical trials [169]. Another extensive library screen of 200,000 small molecules identified compound B02 as a specific inhibitor of RAD51 dependent DNA strand exchange, with no activity on related RAD family and RecA proteins [158]. Further experiments with B02 further characterised its effects in breast cancer cell lines and *in vivo* xenograft models. This showed that B02 enhanced cisplatin sensitivity and disrupted RAD51 foci formation in cell lines. In the xenograft models, combination therapy showed significant decreases in tumour growth compared to treatment with cisplatin alone, with no observable toxicity. Further work with B02 continues to attempt to improve the potency and optimise dosing for incorporation into clinical trials [159]. A recent study showed that quinazolinone derivatives have vastly improved potency over B02 in sensitizing a panel of cell lines to cisplatin, particularly in metastatic and triple negative breast cancer, however their effects *in vivo* have not been tested [170].

While it has not had so much focus as other downstream FA pathway components, preliminary studies have also identified inhibitors of REV7. These have aimed to disrupt the interaction between the REV7 and REV3L subunits of POLζ, thus preventing strand extension in TLS. Due to the unstructured nature of uncomplexed REV7, a structure based rational approach to inhibitor design is difficult. High throughput screening using a competitive binding assay identified 1 inhibitor of the interaction, the potency of which was further improved using structure activity studies. This was confirmed to bind directly to REV7, and functional studies confirmed that it both inhibited ICL repair of reporter plasmids and chemosensitized cancer cells to cisplatin [155]. However, there are concerns regarding the toxicity of the drug in the absence of cisplatin, and further studies of the mechanism of action are required before this lead can be progressed.

While targeted therapies against the FA pathway would be a useful approach to improving the efficacy of chemotherapy, particularly in cancers in which the FA pathway has been linked to development of resistance to standard of care therapies such as ovarian [7], glioblastoma [171], myeloma [172] and head and neck [173], more work is required before those which have been discovered so far can be brought into the clinic. Beyond initial identification and development of inhibitors, there is a surprising lack of studies aiming to further characterise these. In particular, a greater mechanistic understanding of many of the broad spectrum inhibitors available, and more emphasis on preclinical testing will help drive the incorporation of these into future clinical trials.

## Synthetic lethality

Due to the complex interplay between the DNA repair pathways, another approach to designing targeted therapies of the FA pathway is to use a synthetic lethality approach. By exploiting pathway defects already present in the tumour, it is possible to induce cell death selectively in tumour cells, while other cells without pathway defects are unaffected. The most well-known example of a successful synthetic lethality approach is the case of PARP inhibitors as ovarian cancer treatment, particularly in the case of BRCA1/2 deficient cells, which are defective in both HR and ICL repair [174]. While HR in particular has been a focus for synthetic lethality therapies and is promoted by the FA pathway [175], FA proteins are involved in a number of different DNA repair pathways besides ICL and HR, broadening the therapeutic opportunities available. For example, REV7 has been shown to inhibit resection of 5’ DNA ends to promote NHEJ [176, 177], and FANCA is a key factor in DSB repair by single strand annealing [178]. There may therefore be advantages to studying this pathway in the wider context for synthetic lethality approaches, to maximize therapeutic potential.

### PARP inhibition

PARP inhibition is synthetic lethal with BRCA1/2 loss of function mutations, as it prevents the repair of single strand breaks by PARP and the base excision repair (BER) pathway, causing frequent DNA double strand breaks. These require repair by the defective homologous repair pathway, and if they persist lead to chromosomal instability and apoptosis [174]. Several PARP inhibitors are currently approved for use in ovarian cancer patients following successful clinical trials. Olaparib has been approved as maintenance therapy following first line treatment for *BRCA1/2* mutated ovarian cancers in the EU and USA since 2014 [179]. Several trials in man have shown olaparib to significantly improve progression free survival (PFS), particularly in *BRCA1/2* mutated patients [180, 181]. The most recent phase III trial of olaparib demonstrated even more dramatic results, with risk of death or disease progression reduced by 70% when chemotherapy is followed with olaparib maintenance treatment in patients with *BRCA1/2* mutations [76]. Another PARP inhibitor, niraparib, was approved for use in platinum sensitive, recurrent ovarian cancer in 2017 [182]. A phase III trial showed significant improvements in PFS for ovarian cancer patients treated with niraparib across two cohorts of patients, interestingly both with and without BRCA mutations and HR deficiencies in contrast to the olaparib trial [183]. A third inhibitor, rucaparib, again demonstrated improvements in PFS regardless of *BRCA* mutation and HR deficiency status in ovarian cancer, and was also approved for treatment of BRCA mutant ovarian cancers in 2017 [184, 185]. This indicates that other factors may affect PARPi sensitivity, and it would be interesting to study the context of other repair pathway deficiencies, including the FA pathway, on PARPi sensitivity to further elucidate strategies for stratifying patients.

It has been shown that the synthetic lethality effect of PARPis is not limited to BRCA1/2, or indeed to the HR proteins that function in ICL. Knockdown of RAD51, FANCD2, FANCA or FANCC have all been shown to sensitise fibroblasts to PARP inhibition [81], demonstrating that PARPis may have wider applicability, and the FA proteins may be useful as patient stratification biomarkers. It is of particular importance that the effectiveness of PARP treatment was not limited to HR protein knockdown, and was also observed when the classical FA proteins were supressed, demonstrating that this phenomenon is not merely a result of the HR function of BRCA1/2. The effect of RAD51C silencing on PARP inhibitor treatment in cancer cell lines and xenografts was also investigated, and was found to significantly sensitize tumours to treatment and inhibit tumour growth [186]. A more recent clinical trial attempted to elucidate the roles of mutations in the FA genes on PARPi treatment outcomes in ovarian cancer patients [187]. However, although mutations were observed, notably in *FANCJ*, *FANCA*, *FANCD2*, *FANCL*, *RAD51* and *RAD51C*, these were of very low frequency, and it was therefore difficult to achieve significance in their findings. Improvements in PFS were however observed for the group of patients with DNA repair pathway defects, although this also included those with defects in other repair pathways than FA [187]. More emphasis on those patients without mutations, but which still have deficiencies in FA protein expression, may improve the scope of such trials, particularly in the ovarian cancer landscape, in which driver mutations can be rare.

### Beyond PARP inhibition

Aside from the obvious potential of PARP inhibitors extending to FA deficient cells beyond *BRCA1/2* mutants, other inhibitors have also begun to be investigated for their synthetic lethality with the FA pathway. Initial screening of drug and compound libraries in cell lines proficient and deficient for the FA pathway has shown that it is possible to use high throughput screening to identify compounds to which FA deficient cells are hypersensitive, and do not function by inducing ICLs [188]. Another screen used a siRNA knockdown approach in isogenic cell lines with and without FA defects. This confirmed that knockdown of PARP and other proteins of the BER pathway was synthetic lethal with FA deficiency, but also identified the ataxia telangiectasia mutated (ATM) mediated double strand break repair pathway as synthetic lethal with the FA pathway. This was confirmed using ATM inhibiting drugs, which demonstrates a promising new utility for ATM inhibitors in cancer therapy [189].

Another screen used an opposite approach to discover targets that showed synthetic lethality with WEE1 inhibitors in colon cancer cell lines, which identified several FA proteins. This was postulated to be due to the role that they play in replicative stress, rather than the ICL repair pathway itself [190]. A later study however gave contradictory results, with FA proficient pancreatic cancer cells observed to have higher sensitivity to WEE1 inhibition. Therefore, it was proposed that this synthetic lethality may be dependent on cell lineage and other genetic factors altering the cellular context [191]. This highlights the importance of considering other external factors and using a variety of models in inhibitor development, particularly in the case of synthetic lethality, as these can have dramatic changes on results.

A more rational, function driven approach was taken by another study, which aimed to characterise the effect of CHK1 inhibition on cells with FA deficiencies. CHK1 controls the G2/M checkpoint which is hyperactivated in FA patient cells, leading to accumulation in G2 phase and allowing repair of ICL damage by other pathways prior to mitosis, thus enabling cell survival. It was therefore hypothesized that FA pathway deficient tumours were “addicted” to this checkpoint, and knockout would cause synthetic lethality. Indeed, knockdown of CHK1 by both siRNA and inhibitors in cell lines and zebrafish models showed that this was the case. Interestingly, the combination of CHK1 inhibitor and FA deficiency also hypersensitized cells to cisplatin, more so than FA deficient cells untreated with CHK1 inhibitor [192]. This provides an elegant combination of the chemosensitization and synthetic lethality approaches, which may be further utilised in future.

## Conclusions

The development of FA targeting inhibitors is still at an early stage and the majority of drugs identified to date are likely to be relatively non-specific for the pathway; however there is ongoing interest in seeking to identify more specific inhibitors. It will be important to assess the interaction of newly identified inhibitors with cisplatin or carboplatin to assess their efficacy in selected cancer groups. A number of inhibitors have already been identified that synergise with either cisplatin or carboplatin supporting this approach. In addition to further inhibitor development, studies are also required to identify the specific molecular aberrations e.g. mutation, overexpression etc of individual FA proteins that will help select individual cancers as good targets for treatment. The success of the PARP inhibitors in ovarian cancer supports the view that inhibition of DNA repair pathways may have therapeutic value for selected patients particularly when used to sensitise cancers to platinum therapy and generates hope that the FA pathway could provide further useful targets.

## Abbreviations

ATM: ataxia telangiectasia mutated
BER: base excision repair
DDN: 2,3-dichloro-5,8-dihydroxy-1,4-naphthoquinone
DSB: double strand break
FA: Fanconi anaemia
FAAP: Fanconi anaemia associated protein
FEN1: flap endonuclease 1
HR: homologous recombination
ICL: interstrand crosslink
MMC: mitomycin C
NER: nucleotide excision repair
PARP: poly ADP ribose polymerase
PARPi: poly ADP ribose polymerase inhibitor
PFS: progression free survival
RMI: RecQ-mediated genome instability protein
TLS: translesion synthesis

## Declarations

### Copyright

© The Author(s) 2020.
